# 25 Enteral Acetaminophen Induced Hypotension: Getting It Right vs Being Right

**DOI:** 10.1093/jbcr/irae036.025

**Published:** 2024-04-17

**Authors:** Natalie V Kesler, Curt C Bay, Brook Chavarria, Elizabeth Thorstenson, Asia N Quan, Nisha Talanki, Van Dobbe, Suzanne C Osborn, Claudia Islas, Tiffany Hockenberry, Karen J Richey, Kevin N Foster

**Affiliations:** ValleyWise Health Medical Center, Peoria, Arizona; A.T. Still University, Mesa, AZ; Arizona Burn Center Valleywise Health, Phoenix, Arizona; Valleywise Health, Rio Verde, Arizona; Arizona Burn Center, Phoenix, AZ; Valleywise Health, Phoenix, AZ; ValleyWise Health Medical Center, Peoria, Arizona; A.T. Still University, Mesa, AZ; Arizona Burn Center Valleywise Health, Phoenix, Arizona; Valleywise Health, Rio Verde, Arizona; Arizona Burn Center, Phoenix, AZ; Valleywise Health, Phoenix, AZ; ValleyWise Health Medical Center, Peoria, Arizona; A.T. Still University, Mesa, AZ; Arizona Burn Center Valleywise Health, Phoenix, Arizona; Valleywise Health, Rio Verde, Arizona; Arizona Burn Center, Phoenix, AZ; Valleywise Health, Phoenix, AZ; ValleyWise Health Medical Center, Peoria, Arizona; A.T. Still University, Mesa, AZ; Arizona Burn Center Valleywise Health, Phoenix, Arizona; Valleywise Health, Rio Verde, Arizona; Arizona Burn Center, Phoenix, AZ; Valleywise Health, Phoenix, AZ; ValleyWise Health Medical Center, Peoria, Arizona; A.T. Still University, Mesa, AZ; Arizona Burn Center Valleywise Health, Phoenix, Arizona; Valleywise Health, Rio Verde, Arizona; Arizona Burn Center, Phoenix, AZ; Valleywise Health, Phoenix, AZ; ValleyWise Health Medical Center, Peoria, Arizona; A.T. Still University, Mesa, AZ; Arizona Burn Center Valleywise Health, Phoenix, Arizona; Valleywise Health, Rio Verde, Arizona; Arizona Burn Center, Phoenix, AZ; Valleywise Health, Phoenix, AZ; ValleyWise Health Medical Center, Peoria, Arizona; A.T. Still University, Mesa, AZ; Arizona Burn Center Valleywise Health, Phoenix, Arizona; Valleywise Health, Rio Verde, Arizona; Arizona Burn Center, Phoenix, AZ; Valleywise Health, Phoenix, AZ; ValleyWise Health Medical Center, Peoria, Arizona; A.T. Still University, Mesa, AZ; Arizona Burn Center Valleywise Health, Phoenix, Arizona; Valleywise Health, Rio Verde, Arizona; Arizona Burn Center, Phoenix, AZ; Valleywise Health, Phoenix, AZ; ValleyWise Health Medical Center, Peoria, Arizona; A.T. Still University, Mesa, AZ; Arizona Burn Center Valleywise Health, Phoenix, Arizona; Valleywise Health, Rio Verde, Arizona; Arizona Burn Center, Phoenix, AZ; Valleywise Health, Phoenix, AZ; ValleyWise Health Medical Center, Peoria, Arizona; A.T. Still University, Mesa, AZ; Arizona Burn Center Valleywise Health, Phoenix, Arizona; Valleywise Health, Rio Verde, Arizona; Arizona Burn Center, Phoenix, AZ; Valleywise Health, Phoenix, AZ; ValleyWise Health Medical Center, Peoria, Arizona; A.T. Still University, Mesa, AZ; Arizona Burn Center Valleywise Health, Phoenix, Arizona; Valleywise Health, Rio Verde, Arizona; Arizona Burn Center, Phoenix, AZ; Valleywise Health, Phoenix, AZ; ValleyWise Health Medical Center, Peoria, Arizona; A.T. Still University, Mesa, AZ; Arizona Burn Center Valleywise Health, Phoenix, Arizona; Valleywise Health, Rio Verde, Arizona; Arizona Burn Center, Phoenix, AZ; Valleywise Health, Phoenix, AZ

## Abstract

**Introduction:**

Fevers in burn injured patients is a common phenomenon and is typically managed with enteral acetaminophen (APAP) in our center. While hypotension is a known side effect of IV APAP, it has not been reported with enteral administration. However, burn nurses report episodes of hypotension in critically ill patients following APAP that are reproducible and report hesitancy to give additional doses, fearing a cause-and effect relationship. Conversely, the medical team views the relationship as temporal in nature and believes continued use of APAP an appropriate therapy to treat hyperthermia. The purpose of this study was to examine the relationship between administration of enteral APAP and hypotension in critically ill patients with thermal burns ≥ 20% total body surface area (TBSA).

**Methods:**

This was a retrospective chart review of patients over a 5-year period. Primary outcome measures were number of patients receiving oral APAP, incidence of patients experiencing a drop in systolic blood pressure (SBP) of 20 points mmHg and/or a 15% decrease from baseline, within 3 hours, and number of qualifying hypotensive events. Descriptive statistics and Pearson correlation were calculated.

**Results:**

A total of 203 patients suffered a burn injury of ≥ 20% TBSA during the study period. Among those 196 received enteral Tylenol, 180 (92%) experienced a hypotensive event as defined in the protocol. After consultation with a biostatistician and burn surgeon, hypotension was redefined as a SBP ≤ 100 mmHg. Using this definition 78 (40%) patients experienced at least one hypotensive event within 3 hours of APAP administration with a mean SBP of 88 mmHg, (range 58-100 mmHg). There was a positive correlation between hypotension and %TBSA (p < .001), baseline SBP (p < .000), baseline temp (p=.007), bilirubin (p < .001), mechanical ventilation (p < .001), acute adrenal insufficiency (p=.036), compartment syndrome (p=.02), and ventilator associated pneumonia (p < .005). There was no correlation between hypotension and APAP.

**Conclusions:**

Based on our data, larger TBSA, mechanical ventilation, ventilator associated pneumonia, lower baseline SBP, increased temperature, adrenal insufficiency and compartment syndrome showed an increase in probability of a hypotensive event occurring. However, despite strong convictions from bedside nurses the study did not demonstrate a significant relationship between hypotension and enteral APAP administration. A prospective, observational study is warranted to further examine this phenomenon.

**Applicability of Research to Practice:**

Enteral APAP can be given by bedside nursing to control hyperthermia in critically ill burn patients without hesitation or fear of causing hypotension.

